# Supplementary material for: Mistreatment of Women during Childbirth and Associated Factors in Northern West Bank, Palestine
Source: Int J Environ Res Public Health. 2022 Oct 13;19(20):13180. doi: 10.3390/ijerph192013180 (PMC9602547; doi:10.3390/ijerph192013180)
Supplement: Supplementary file 1 [file ijerph-19-13180-s001.zip › File S1-The questionnaire.pdf]

The questionnaire

القسم الأول
Section one

المعلومات الديموغرافية
Socio- demographic data

العمر (......................)
Age

المستوي التعليمي
Level of education

أساسي ☐ ثانوي ☐ دبلوم متوسط ☐ بكلوريوس ☐
Primary | High school | Diploma / 2 years | Bachelor /4 years
غير ذلك ☐

Others

هل لديكِ عمل؟
Are you employed?

لا ☐ نعم ☐ ماهي طبيعة عملك (......................)
No | Yes | ***What is your work؟***

مكان السكن
Residential location

مدينة ☐ قرية ☐ مخيم ☐ غير ذلك ☐
City | Village | Camp | Others

معدل دخل الأسرة الشهري (......................)
Monthly family income

هل لديك تامين صحي ؟
Do you have health insurance?

نعم ☐ ، مصدر التأمين (......................) لا ☐
Yes

سِجلّ حالات الحمِل والولادات
Labour and delivery characteristics

عدد الوِلادات - التي تمت بعد 24 اسبوع (......................)
No. of deliveries without abortion

مقدِّم الرعاية الصحية، الذي أشرف على رعايتك أثناء فترة المخاض والولادة (يمكن أن تشيري إلى أكثر من خيار)
The health care providers who followed the current labour (You can choose more than one)

قابله قانونية ☐ طبيبة نسائية ☐ طبيب نسائي ☐ غير ذلك ☐ (..........)
Midwife Female doctor Male doctor Others

الساعة التي تمت بها الولادةالاخيرة (.......................)
Time of delivery of the last delivery

عدد الساعات التي استغرقتها فترة المخاض والولادة الاخيرة (.......................)
Duration of current childbirth by hours

عملية الولاده الاخيرة تمت بشكل
Your labour started as
تلقائي ☐ تحفيز باستخدم طلق صناعي ☐
Spontaneous Augmentation

هل تلقيتِ مُسكّن للألم خلال مرحلة المخاض والولادة الاخيرة؟
Did you receive any pain management during childbirth?
نعم ☐ لا ☐
Yes No

طريقة الولادة الأخيرة
Mode of your last delivery:

ولادة طبيعية دون الحاجه لشق جراحي مهبلي/جرح ☐
Vaginal delivery without episiotomy/tear

ولادة طبيعية مع الحاجة لشق جراحي مهبلي/ جرح ☐
Vaginal delivery with episiotomy/tear

نتيجة الولادة الأخيرة
Outcome of your childbirth:

ذكر حي ☐ أنثى حية ☐
Alive male Alive female

هل طرأ أيّة مضاعفات خلال الولادة الأخيرة
Did you face any complication during childbirth?

نعم - مضاعفات لي ☐ نعم - مضاعفات لطفلي ☐
Yes- complication to me Yes- complication to my baby

نعم- مضاعفات لي و لطفلي ☐ لا - لم يطرأ أيّة مضاعفات ☐
Yes- complication to me and to my baby No- no complications happened

المستشفى الذي تمّت فيه الولادة الأخيرة
The facility of your childbirth was:

حكومي ☐ خاص ☐
Governmental hospital Nongovernmental hospital

مقدّم الرعاية الصحية الذي قام بأجراء الولادة الأخيرة
The health care providers who conducting your last delivery

قابله قاتونبة ☐ طبيبة نسائية ☐

Midwife Female doctor

طبيب نسائي ☐ غير ذلك ☐ (......................)

Male doctor Others

الفترة الزمنية من بعد الولادة حتى ايوم (......................)

How long have you given birth

القسم الثاني

Section Two

الرضا عن الرعاية الصحية المقدمة أثناء فترة المخاض والولادة

Satisfaction of care during childbirth

السلوك الإيجابي للقابلة / الممرضة والبيئة المادية

Positive behaviour of midwife/nurse and physical environment

لا أوافق بشدة Strongly disagree	لا أوافق disagree	لا ينطبق	أوافق Agree	أوافق بشدة Strongly agree	البند Statement
					كانت القابلة /الممرضة تعاملني بلطف أثناء فترة المخاض والولادة<br>*The midwife/nurse treated me kindly during childbirth*
					كانت القابلة / الممرضة متعاونه معي أثناء فترة المخاض والولادة<br>*The midwife/nurse was cooperative during childbirth*
					كانت القابلة / الممرضة تشجعني أثناء فترة المخاض والولادة<br>*The midwife/nurse was encouraging me during childbirth*
					كانت غرفة الولادة نظيفة<br>*Labor room was clean*

السلوك الإيجابي للطبيب والدعم المعلوماتي

Positive behaviour of physician and informational support

لا أوافق بشدة Strongly disagree	لا أوافق disagree	لا ينطبق	أوافق Agree	أوافق يشدة Strongly agree	البند Statement
					كان الطبيب يعاملني بلطف أثناء فترة المخاض والولادة<br>*The doctor treated me kindly during childbirth*
					كان الطبيب متعاوناً معي أثناء فترة المخاض والولادة<br>*The doctor was cooperative during childbirth*
					كان الطبيب يشجعني أثناء فترة المخاض والولادة<br>*The doctor was encouraging me during childbirth*
					زودتني القابلة / الممرضة بالمعلومات اللازمة عن المخاض والولادة<br>*The midwife/nurse provided me with all information needed during childbirth*
					زودني الطبيب بالمعلومات اللازمة عن المخاض والولادة<br>*The doctor provided me with all information needed during childbirth*
					قدم الطبيب لي ألإرشادات اللازمة لوضعي<br>The doctor provided a specified teaching for related to my condition during childbirth

القسم الثالث

جودة الرعاية الصحية المقدمة أثناء فترة المخاض والولادة

Perceived quality of care during childbirth

الرعاية المقدمة من قبل الطبيب

Care provided by physician

لا أوافق بشدة Strongly disagree	لا أوافق disagree	لا ينطبق	أوافق Agree	أوافق يشدة Strongly agree	البند Statement
					كان عدد الأطباء في غرفة الولادة كافيا لتقديم الرعاية اللازمة<br>*Doctors were adequate to monitor labor and childbirth*
					يمتلك الطبيب الكفاءة اللازمة للعناية بي أثناء فترة المخاض والولادة<br>*The doctor was competent to care for me*
					أتاح لي الطبيب الفرصة للتعبيرعن مشاعري بشكل مريح<br>*The doctor allowed me to freely expressing my feelings during childbirth*

					كان الوقت المخصص للعناية بي من قبل الطبيب كافياً<br>*The time which was devoted by the doctor was adequate to care for me*
					حافظ الطبيب على خصوصيتي أثناء فترة المخاض والولادة<br>*My privacy was protected by the doctor during childbirth*
					تعامل الطبيب معي باحترام أثناء فترة المخاض والولادة<br>*The doctor treated me with respect*
					كان الطبيب يلبّي جميع احتياجاتي الطبية دون تقصير اثناء فترة المخاض والولادة<br>*The doctor would fulfil all my needs without default*
					كان الطبيب يتعامل بهدوء مع ردّات فعلي العصبية أثناء فترة المخاض والولادة<br>*The doctor was calmly dealing with my nervous reaction*

الرعاية المقدمة من قبل القابلة/ الممرضة
Care provided by midwife/nurse

لا أوافق بشدة **Strongly disagree**	لا أوافق **disagree**	لا ينطبق	أوافق **Agree**	أوافق يشدة **Strongly agree**	البند **Statement**
					كان عدد القابلات في غرفة الولادة كافيا لتقديم الرعاية اللازمة<br>*Midwife/nurses were adequate to monitor labor and childbirth*
					تمتلك القابلة الكفاءة اللازمة للعناية بي أثناء فترة المخاض والولادة<br>*The midwife/nurse was competent to care for me*
					أتاحت لي القابلة / الممرضه الفرصة للتعبيرعن مشاعري بشكل مريح<br>*The midwife/nurse allowed me to freely expressing my feelings during childbirth*
					كان الوقت المخصص للعناية بي من قبل القابلة / الممرضة كافياً<br>*The time which was devoted by the midwife/nurse was adequate time to care for me*
					حافظت القابلة / الممرضة على خصوصيتي أثناء فترة المخاض والولادة<br>*My privacy was protected by the midwife/nurse during childbirth*
					تعاملت القابلة معي باحترام أثناء فترة المخاض والولادة<br>*The midwife/nurse treated me with respect*
					كانت القابلة / الممرضة تلبّي جميع احتياجاتي دون تقصير أثناء فترة المخاض والولادة

					The midwife/nurse would fulfil all my needs without default
					كانت القابلة / الممرضة تتعامل بهدوء مع ردّات فعلي العصبية أثناء فترة المخاض والولادة<br>*The midwife/nurse was calmly dealing with my nervous reaction*

القسم الرابع

Section 4

انواع التعامل التي تلقّتها السيدة أثناء فترة المخاض والولادة

التعامل الجسديّ أثناء فترة المخاض والولادة

Physical abuse

لا ينطبق<br>Not applicable	لا<br>No	نعم<br>Yes	البند<br>Statement
			أجلستُ بوضع غيرمريح اثناء فترة المخاض والولادة<br>*I was left in uncomfortable position during childbirth*
			كان الفحص الداخلي لقياس عنق الرحم مؤلماً أثناء فترة المخاض والولادة<br>*Vaginal examination done to me was painful*
			ضربت القابلة / الممرضة على فخديّ /وجهي أثناء الولادة<br>*I encountered slapping on my thigh/face during childbirth by the midwife*
			ضرب الطبيب على فخديّ /وجهي أثناء المخاض والولادة<br>*I encountered slapping on my thigh/face during childbirth by the doctor*
			تعرضتُ للضغط على البطن اثناء الولادة<br>*I subjected to abdominal pressure during childbirth*

التعامل اللّفظي أثناء فترة المخاض والولادةVerbal abuse

لا ينطبق<br>Not applicable	لا<br>No	نعم<br>Yes	البند<br>Statement
			أجابت القابلة او الممرضة على استفساراتي بطريقة قاسية اثناء فترة المخاض والولادة<br>*The midwife/nurse replied to me in a tough way during childbirth*
			أجاب الطبيب على استفساراتي بطريقة قاسية اثناء فترة اللمخاض والولادة<br>*The doctor replied to me in a tough way during childbirth*
			وُبِّختُ من قبل القابلة أثناء فترة المخاض والولادة<br>*I was scolded by the midwife/nurse during childbirth*
			وُبِّختُ من قبل الطبيب أثناء فترة المخاض والولادة<br>*I was scolded by the doctor during childbirth*

آلية التعامل أثناء المخاض والولادة

Stigma and discrimination

لا ينطبق<br>Not applicable	لا<br>No	نعم<br>Yes	البند<br>Statement
			لامتني القابلة/ الممرضة بسبب عُمري<br>*I was blamed by the midwife/nurse because of my age during childbirth*
			لامني الطبيب بسبب عُمري<br>*I was blamed by the doctor because of my age during childbirth*
			لامتني القابلة / الممرضة على تعدد حالات ولاداتي لمّا تألمْتُ<br>*I was blamed by the midwife/nurse during labor pain because I had had multiple deliveries*
			لامني الطبيب على تعدّد حالات ولاداتي لمّا تألمْتُ<br>*I was blamed by the doctor during labor pain because I had had multiple deliveries*
			أجّلت القابلة التعامل مع حالتي، بحجّة أنها كانت ولادتي الأولى<br>*I faced delaying of care from the midwife because it was my first delivery*
			أجّل الطبيب التعامل مع حالتي،بحجّة أنها كانت ولادتي الأول<br>*I faced delaying of care from the doctor because it was my first delivery*

التعامل المهني اثناء فترة المخاض والولادة:
الشرح الواضح لبعض الإجراءات الطبية والموافقة عليها

Failure to meet professional standard - Lack of informed consent and explanation

لا ينطبق<br>Not applicable	لا<br>No	نعم<br>Yes	البند<br>Statement
			قامت القابلة/ الممرضة بعمل بعض الإجراءآت لي دون اخذ موافقتي على ذلك<br>*Some procedure was done by the midwife/nurse without consent*
			قام الطبيب بعمل بعض الإجراءآت لي دون اخذ موافقتي على ذلك<br>*Some procedure was done to me by doctor without consent*
			قامت القابلة / الممرضة بشرح تفاصيل أي فحص / تدخل طبي يتطلب مني الموافقه عليه<br>*The midwife/nurse explained to me any medical intervention or examination that requires my approval*
			قام الطبيب بشرح تفاصيل أي فحص / تدخل طبي يتطلب مني الموافقه عليه<br>*The doctor explained to me any medical intervention or examination that requires my approval*

Failure to meet professional standard - Negligence of care during childbirth
السرّية في التعامل أثناء فترة المخاض والولادة

لا ينطبق<br>Not applicable	لا<br>No	نعم<br>Yes	البند<br>Statement
			قامت القابلة / الممرضة بمناقشة بعض الأمور التي تخصني على مسمع الآخرين<br>*The midwife/nurse discussed my personal history in earshot of other clients*
			قام الطبيب بمناقشة بعض الأمور التي تخصني على مسمع الآخرين<br>*The doctor discussed my personal history in earshot of other clients*
			قامت القابلة /الممرضة بالتصريح عن بعض معلوماتي الصحية على مسمع الآخرين<br>*Health information was disclosed by the midwife/nurse in front of non-health staff*
			قام الطبيب بالتصريح عن بعض معلوماتي الصحية على مسمع الآخرين<br>*Health information was disclosed by the doctor in front of non-health staff*

الرعاية الصحية للسيدة أثناء فترة المخاض والولادة Failure to meet professional standard
Violation of confidentiality during childbirth

لا ينطبق<br>Not applicable	لا<br>No	نعم<br>Yes	البند<br>Statement
			قدمت لي القابلة الاهتمام االلازم أثناء فترة المخاض والولادة

			The midwife/nurse provided me with all the attention that I need during childbirth
			قدم لي الطبيب الاهتمام اللازم أثناء فترة المخاض والولادة<br>*The doctor provided me all the attention that I need during childbirth*
			مرّت فترة من الوقت لم يُفسَّر لي ما يجري من حولي<br>*There were times that no one explained to me what is going on*
			واجهتُ بعض التاخير في الرعاية الصحية التي قدمت لي من قبل القابلة اثناء فترة المخاض والولادة<br>*I encountered delay of care from the midwife/nurse*
			واجهتُ بعض التاخير في الرعاية الصحية التي قدمت لي من قبل الطبيب اثناء فترة المخاض والولادة<br>*I encountered delay of care from the doctor*

العلاقة بين مقدّمي الرعاية الصحية والسيدة أثناء فترة المخاض والولادة:
التواصل بين السيدة ومقدمي الرعاية الصحية أثناء فترة المخاض والولادة

Poor rapport between women and providers-Ineffective communication during childbirth

لا ينطبق<br>Not applicable	لا<br>No	نعم<br>Yes	البند<br>Statement
			عرّفتني القابلة / الممرضه التي قامت على رعايتي عن نفسها قبل البدء بفحصي<br>*I was kept known the name and the identity of the midwife/nurse dealing with me during childbirth*
			عرفني الطبيب الذي قام على رعايتي عن نفسه قبل البدء بفحصي<br>*I was kept known the name and the identity of the doctor who cared for me during childbirth*
			لم تكن التفسيرات التي قدمتها لي القابلة / الممرضه أثناء فترة المخاض الولادة كافية<br>*The explanation given by the midwife/nurse during childbirth was not enough*
			لم تكن التفسيرات التي قدمها لي الطبيب أثناء فترة المخاض الولادة كافية<br>*The explanation given by the doctor during childbirth was not enough*

الدّعم المعنوي للسيدة أثناء فترة المخاض والولادة

Poor rapport between women and providers - Lack of supportive care

لا ينطبق<br>Not applicable	لا<br>No	نعم<br>Yes	البند<br>Statement
			لم يسمح بوجود مرافق أثناء فترة المخاض والولادة

			Birth companion was not allowed during childbirth
			كانت القابلة / الممرضه تطمئنني عن ما يحْدث معي أثناء فترة المخاض والولادة<br>*The midwife/nurse was reassuring during childbirth*
			كان الطبيب يطمئني عن ما يحْدث معي أثناء فترة المخاض والولادة<br>*The doctor was reassuring during childbirth*

الاستقلالية للسيدة أثناء فترة المخاض والولادة

Poor rapport between women and providers - Loss of autonomy

**لا ينطبق**<br>**Not applicable**	**لا**<br>**No**	**نعم**<br>**Yes**	**البند**<br>**Statement**
			شاركتُ في اتخاذ القرارات التي تخص العناية بي أثناء فترة المخاض والولادة<br>*I participated in decision making and care provided to me*
			فقدّتُ السيطرة على ما يحث معي أثناء فترة المخاض والولادة<br>*I felt loss of control of what happened during childbirth*
			كنت راضية عن الطريقة التي تَعاملَ بها مقدمو الرعاية الصحية مع جسدي أثناء فترة المخاض والولادة<br>*The way people handling my body was satisfactory*

الخصوصية للسيدةأثناء فترة المخاض والولادة

Health system conditions and constrains - Physical privacy during examination and delivery

**لا ينطبق**<br>**Not applicable**	**لا**<br>**No**	**نعم**<br>**Yes**	**البند**<br>**Statement**
			قامت القابلة بتغطية جسدي جيدًاخلال الفحص الداخلي<br>*The midwife/nurse covered me well during examination*
			قام الطبيب بتغطية جسدي جيداًخلال الفحص الداخلي اوالولادة<br>*The doctor covered me well during examination*
			استخدمت القابلة جميع الوسائل المتاحة للحفاظ على خصوصيتي<br>*The midwife/nurse used all the available resources to protect my privacy*
			استخدم الطبيب جميع الوسائل المتاحة للحفاظ على خصوصيتي<br>*The doctor used all the available resources to protect my privacy*
			كانت المصادر للحفاظ على خصوصيتي متوفرة في غرفة الولادة<br>*The facilities available in labor room are sufficient to maintain my privacy*
