# Supplementary material for: Mistreatment of Women during Childbirth and Associated Factors in Northern West Bank, Palestine
Source: Int J Environ Res Public Health. 2022 Oct 13;19(20):13180. doi: 10.3390/ijerph192013180 (PMC9602547; doi:10.3390/ijerph192013180)
Supplement: Supplementary file 1 [file File S2-The results of multiple logistic regression.pdf › File S2-The results of multiple logistic regression.pdf]

Response to Reviewer 2 – Round 2- Comments

This is the results of multiple logistic regression for each type of mistreatment

The results of multiple logistic regression for each type of mistreatment:

Table S1. Multiple logistic regression analysis for factors associated with physical abuse (n=269)

**Variable**	**Crude OR[a] (95% CI[b])**	***p*- value**	**a.OR[c] (95%CI[b])**	***p*- value**
Age (years)	0.921 (0.869,0 .977)	0.005	0.911 (0.857, 0.969)	0.003
Nature of labor				
Spontaneous	1		1	
Induction	2.603 (1.401, 4.837)	0.002	3.029 (1.581, 5.806)	0.001
Type of facility				
Private	1		1	
Public	1.657 (0.934, 2.940)	0.084	2.174 (1.176, 4.018)	0.013

[a]OR = Odds Ratio
[b]CI = Confidence interval
[c]a.OR = Adjusted Odds Ratio
Forward Likelihood Ratio applied
No multicollinearity and no interaction detected
Hosmer-Lemeshow test, p-value = 0.583
Classification table 76.6% correctly classified
Area under the receiver operating characteristic was 70.5%

Table S2. Multiple logistic regression analysis for factors associated with verbal abuse (n=269)

Variable	Crude OR[a] (95% CI[b])	*p*-value	a.OR[c] (95%CI)	*p*-value
Age	0.933 (0.876, 0.994)	0.031	0.922 (0.864, 0.984)	0.014
Type of facility				
Private	1		1	
Public	0.334 (1.537, 5.841)	0.001	3.254 (1.652 6.412)	0.001

[a]OR = Odds Ratio
[b]CI = Confidence interval
[c]a.OR = Adjusted Odds Ratio
Forward Likelihood Ratio applied
No multicollinearity and no interaction detected
Hosmer-Lemeshow test, p-value =0.357
Classification table 75.5% correctly classified
Area under the receiver operating characteristic was 66.9%

Table S3 Multiple logistic regression analysis for factors associated with stigma and discrimination (n=269)

Variable	Crude OR[a] (95% CI[b])	*p*-value	a.OR[c] (95%CI)	*p*-value
Type of facility				
Private	1		1	
Public	10.393 (2.427, 44.510)	0.002	16.770 (3.787, 74.261)	<0.001
Type of delivery				
Vaginal no episiotomy/tear	1		1	
Vaginal with episiotomy/tear	3.562 (1.580, 8.029)	0.002	5.976 (2.524, 14.153)	<0.001
Residency				
City	1		1	
Village	2.167 (1.001, 4.694)	0.050	2.329 (1.010, 5.370)	0.047

[a]OR = Odds Ratio

[b]CI = Confidence interval

[c]a.OR = Adjusted Odds Ratio

Forward Likelihood Ratio applied

No multicollinearity and no interaction detected

Hosmer-Lemeshow test, p-value =0.806

Classification table 88.1% correctly classified

Area under the receiver operating characteristic was 0.768

Table S4 Multiple logistic regression analysis for factors associated with failure to meet professional standard of care (n=269)

Variable	Crude OR[a] (95% CI[b])	*p*-value	a.OR[c] (95%CI)	P value
Type of facility				
Private	1		1	
Public	3.026 (0.186, 0.587)	<0.001	3.309 (1.833, 5.972)	<0.001
Duration of labor (hour)	1.080 (1.011, 1.154)	0.022	1.099 (1.022, 1.182)	0.011

[a]OR = Odds Ratio

[b]CI = Confidence interval

[c]a.OR = Adjusted Odds Ratio

Forward Likelihood Ratio applied
No multicollinearity and no interaction detected
Hosmer-Lemeshow test, p-value =0.749
Classification table 75.8% correctly classified
Area under the receiver operating characteristic was 0.667

Table S5 Multiple logistic regression for factors associated with poor rapport between women and providers (n=269)

**Variable**	**Crude OR[a] (95% [b]CI)**	***p*-value**	**a.OR[c] (95%CI[c])**	***p*-value**
Type of facility				
Private	1	<0.001	1	
Public	7.451 (3.075, 18.059)		4.612 (1.817, 11.706)	0.001
Education				
Higher than secondary	1	0.001	1	
Secondary or lower	6.102 (2.071, 17.976)		5.092 (1.665, 15.566)	0.004
Receiving pain killer				
Yes	1	0.001	1	
No	5.369 (2.124, 13.567)		3.629 (1.357, 9.708)	0.010

[a]OR = Odds Ratio
[b]CI = Confidence interval
[c]a.OR = Adjusted Odds Ratio
Forward Likelihood Ratio applied
No multicollinearity and no interaction detected
Hosmer-Lemeshow test, p-value =.961
Classification table 88.5% correctly classified
Area under the receiver operating characteristic was 82.7%

Table S6 Multiple logistic regression analysis for factors associated with lack of physical privacy and resources (n=269)

**Variable**	**Crude OR[a] (95%CI[b])**	***p*-value**	**a.OR[c] (95%CI)**	***p*-value**
Type of facility				
Private	1		1	

Public	2.852 (1.431, 5.686)	0.003	2.753 (1.325, 5.683)	0.007
Receiving pain killer	1		1	
Yes	2.180 (1.195, 3.978)	0.011	2.183 (1.130, 4.218)	0.020
No				
Parity	0.847 (0.679, 1.056)	0.140	0.724 (0.565, 0.927)	0.010

[a]OR = Odds Ratio

[b]CI = Confidence interval

a.OR[c] = Adjusted Odds Ratio

Forward Likelihood Ratio applied

No multicollinearity and no interaction detected

Hosmer-Lemeshow test, p-value =0.622

Classficiation table 77.7% correctly classified

Area under the receiver operating characteristic was 63.4%
